# Reference induces biases in late visual processing

**DOI:** 10.1038/s41598-023-44827-8

**Published:** 2023-10-30

**Authors:** Yannan Su, Thomas Wachtler, Zhuanghua Shi

**Affiliations:** 1 Faculty of Biology, Ludwig-Maximilians-Universität München, Munich, Germany; 2 Graduate School of Systemic Neurosciences, Ludwig-Maximilians-Universität München, Munich, Germany; 3 Bernstein Center for Computational Neuroscience, Munich, Germany; 4 General and Experimental Psychology, Ludwig-Maximilians-Universität München, Munich, Germany

**Keywords:** Sensory processing, Neural decoding, Neural encoding, Decision, Perception, Visual system

## Abstract

How we perceive a visual stimulus can be influenced by its surrounding context. For example, the presence of a reference skews the perception of a similar feature in a stimulus, a phenomenon called reference repulsion. Ongoing research so far remains inconclusive regarding the stage of visual information processing where such repulsion occurs. We examined the influence of a reference on late visual processing. We measured the repulsion effect caused by an orientation reference presented after an orientation ensemble stimulus. The participants’ reported orientations were significantly biased away from the post-stimulus reference, displaying typical characteristics of reference repulsion. Moreover, explicit discrimination choices between the reference and the stimulus influenced the magnitudes of repulsion effects, which can be explained by an encoding-decoding model that differentiates the re-weighting of sensory representations in implicit and explicit processes. These results support the notion that reference repulsion may arise at a late decision-related stage of visual processing, where different sensory decoding strategies are employed depending on the specific task.

## Introduction

The world around us is filled with a wealth of visual stimuli, where objects are arranged in a contextual setting. Our perception of the world, thus, is not merely a collection of individual isolated objects, but is susceptible to the surrounding context. This susceptibility to context has been recognized since ancient times, such as the observation in ancient China 400 BC that the moon looks bigger when it rises on the horizon than when it is overhead^1^. Context-based perceptual biases typically appear in the form of repulsion. When two similar objects or features are placed together, they appear more distinct or dissimilar than if presented separately. The repulsion effect has been widely observed in basic visual features, including motion direction^2^, orientation^3,4^, brightness and color^5,6^, numerosity^7^, and even in higher cognitive judgments^8^.

Recent studies have shown that the repulsion bias can be amplified through explicit comparison with an external reference, so-called reference repulsion^9–12^. Jazayeri & Movshon^10^ have demonstrated a classic example of reference repulsion using a dual-task paradigm, where participants reproduced the motion direction of a moving random-dot pattern after comparing it with a reference boundary. In contrast to classical contextual effects such as the tilt effect or the tilt after-effect^3^, the results revealed a systematic bias away from the reference that was strongest when the stimulus aligned with the reference. The authors concluded that the bias in the reproduction task was a result of the first discrimination that caused preferentially weighted signals from neurons tuned away from the reference in decoding. This weighted representation of sensory likelihoods ’repels’ from the reference, resulting in better discrimination^10^. An alternative account proposes that the repulsion bias arises from the sensory encoding rather than the decoding process^13^. It posits that the encoding precision of measurement varies according to the difference between the stimulus and the reference. During decoding, this variable-precision encoding is integrated with a uniform prior to form a posterior distribution^13^. The prediction based on that posterior distribution could produce a similar reference repulsion effect. Both accounts, despite applying different underlying mechanisms, concur in that the reference repulsion originates from the perceptual stage.

Several recent studies, however, have challenged the notion that reference repulsion is solely a perceptual process. For instance, Zamboni et al.^11^ have shown that the presence of the reference during the reproduction task is crucial in eliciting the repulsion effect. When the reference orientation shifted slightly ($\pm {6}^\circ$) during the reproduction task, the repulsive bias also shifted accordingly with the reference. Similarly, Fritsche & de Lange^14^ let participants first judge whether a grating stimulus was clockwise or counterclockwise relative to a previously presented reference boundary. Subsequently, participants indicated whether the stimulus had the same orientation as a comparison stimulus. The researchers found that the perceptual bias that occurred in a successive comparison task had distinct characteristics from those of the reference repulsion bias, suggesting that reference repulsion does not directly alter the appearance of the stimulus, but acts at a late decision stage. This idea also aligns with the self-consistent Bayesian observer model^12,15^, which posits that an optimal observer seeks self-consistency in representations across the hierarchy of inference. In a series of tasks, the decision in one task is influenced by the preceding task, and a prior categorical judgment biases downstream processes, such as reproduction, based on working memory^12,15^. These findings suggest that the repulsion effect may emerge at the late decision stage.

Despite the ongoing debate surrounding the processing stage of the reference repulsion continues, most studies commonly used the reference throughout the trial, starting before or concurrently with the target stimulus. However, the timing of the reference plays a crucial role in determining the processing stage involved in the reference repulsion effect. For example, by presenting the reference after the sensory encoding of the stimulus, early encoding of the reference can be effectively avoided.

Another crucial factor influencing the repulsion effect is the distinction between explicit and implicit processes^16^. Many studies investigating reference repulsion employ an explicit discrimination task before the primary measurement^10,12^. In some cases, participants are exposed to an explicit categorical discrimination task, even if they are not asked to make explicit judgments^9,11,13,14^, potentially causing the categorical decision to influence the main task. Therefore, it is crucial to directly compare the repulsion effect under explicit and implicit instructions to gain deeper insights into the nature of reference repulsion.

On this ground, we conducted a study using an ensemble orientation averaging paradigm to investigate the impact of the reference orientation and task conditions on the repulsion effect. Notably, we presented the reference after the stimulus. Our reasoning was twofold: if reference repulsion primarily occurs during early sensory processing, a post-stimulus reference would have no influence on the observer’s judgment; however, if the reference does affect judgment, we would observe judgment biased away from the reference. In addition, we introduced different task conditions to assess the impact of explicit and implicit processes on the repulsion effect directly.

We found that the post-stimulus reference indeed induced repulsion, but the effect differed between explicit and implicit processes. We attribute the findings to variations in weighting between implicit and explicit processes within an encoding-decoding model.

## Methods

### Participants

Five volunteers, one male and four females, ranging from 21 to 26 years old, participated in the experiments. All participants had normal or corrected-to-normal vision and were right-handed and naive with respect to the purpose of the experiment. Participants signed informed consent prior to the experiment and received a compensation of 10 Euros per hour. The study was approved by the ethics committee of the LMU Munich and carried out in accordance with the Declaration of Helsinki.

### Stimuli

All visual stimuli were generated using the software Psychopy 2020.1.2 [RRID:SCR_006571, Ref.^17^] based on Python 3.7, presented on a ViewPixx Lite 2000A display, with a resolution of $1920 \times 1200$ pixels at a refresh rate of 120 Hz, controlled by a Radeon Pro WX 5100 graphics card.

**Figure 1:**
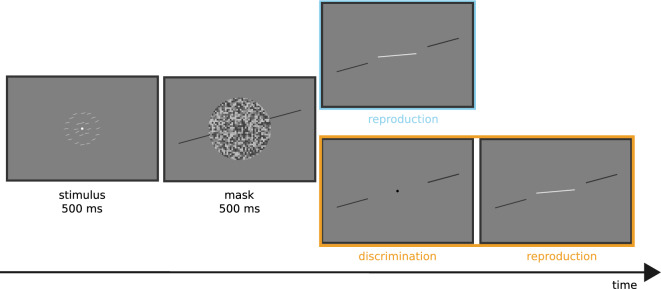
Experiment paradigm. A 500-ms presentation of the ensemble display was followed by a 500-ms circular white noise mask. A reference was presented simultaneously with the mask display and lasted until the end of the trial. Observers had to reproduce the mean orientation of the ensemble with a computer mouse, with (the dual-task blocks, colored orange) or without (the single-task blocks, colored blue) a preceding discrimination task. The task required them to indicate whether the mean orientation was CW or CCW of the reference orientation.

All stimuli were presented on a neutral gray background (106.7 cd/m^2^). The fixation dot (radius ${0.6}^\circ$), if presented, was always shown at the screen center (Fig. 1). A reference consisted of a pair of line segments (length: ${5}^\circ$, line width: ${0.074}^\circ$), both positioned ${5}^\circ$ from the screen center and were colinear, indicating a reference orientation. The ensemble display consisted of 24 tilted bars (length ${0.6}^\circ$, width ${0.1}^\circ$), arranged on a grid of two concentric circles, 8 bars positioned on an inner circle with a diameter of ${1.0}^\circ$, 16 bars positioned on an outer circle with a diameter of ${2.5}^\circ$. Small independent variations were applied to the positions of the bars by adding random shift values (sampled from ${\mathcal {N}}(0, {0.1}^\circ )$) on x-y coordinates. The mask display was a circular patch of white noise (contrast 0.5, spatial frequency 0.03 c/deg). The mask display was positioned at the screen center, with a diameter of ${10}^\circ$ for masking the entire ensemble stimuli, or a diameter of ${15}^\circ$ for masking the entire display after the trial.

The reference orientations were randomly chosen from ${15}^\circ$, ${45}^\circ$, ${75}^\circ$, ${105}^\circ$, ${135}^\circ$, and ${165}^\circ$ (note the ${0}^\circ$ is the vertical). The averaged orientation of the tilted bars was randomly probed around the corresponding reference orientation, with a step of ${3}^\circ$ in a range of [${-18}^\circ$, ${18}^\circ$]. The variation of the orientations within the ensemble display had two versions: a low-noise version, in which all bars had the same orientation (i.e., ${0}^\circ$ of orientation noise), and a high-noise version, in which the orientations randomly varied according to a normal distribution with a standard deviation of ${9}^\circ$.

### Procedures

During the experiment, the subject sat in a dimly lit room and viewed the display binocularly from a distance of 57 cm. The fixation dot was present during the stimulus presentation and response waiting period. Participants were instructed to maintain their eyes on the fixation dot during the trial.

A trial started with a white fixation dot, shown in the center of the screen for 500 ms. Then, an ensemble display with 24 tilted bars appeared for 500 ms, followed by a 500-ms mask display with circular white noise, preventing any afterimage effects. After the ensemble display vanished, a reference was presented simultaneously with the mask display, remaining visible until the final response.

Participants were given two types of block-wise tasks: a single-task and a dual-task. To minimize explicitly using the reference in the single-task condition, the dual-task condition was introduced only after participants completed all required single-task blocks. The single task involved an orientation reproduction, where participants adjusted the orientation of a white line (length ${3.0}^\circ$, initial orientation randomly chosen from ${0}^\circ$ to ${180}^\circ$) at the screen center to indicate the perceived average orientation of the ensemble bars. By moving a computer mouse up or down, they could adjust the line’s orientation. Participants had to confirm the final orientation judgment by pressing the space key. Following the response, a mask display appeared for 500 ms to minimize cross-trial carryover effects.

The dual-task consisted of the orientation reproduction from the single task and a discrimination task. In the discrimination task, participants had to judge whether the averaged orientation was clockwise (CW) or counterclockwise (CCW) relative to the reference orientation by pressing the left or right key of the mouse. The discrimination task was conducted before the reproduction task, with a black fixation dot indicating the discrimination task. If the response time exceeded 4 seconds for discrimination or 8 seconds for reproduction, the corresponding trial was discarded and repeated at the end of the same block. Discarded trials were rare, on average 0.43%. The average response time was $2.04 \pm 0.96$ seconds.

Each block consisted of 156 trials, resulting from the full combination of the reference orientations (6 levels), the corresponding stimulus orientations (13 levels per reference orientation), and the two noise levels (high vs. low). The combination was randomized within each block. In total, there were ten blocks: five blocks for the single task, and five blocks for the dual task, yielding 1560 valid trials in total. Before the main experiment, participants completed a practice block (156 trials) with feedback texts showing the error value of the reproduction (single-task) or the correctness of the discrimination judgment (dual-task).

### Data analysis

Data analysis was performed for individual participants’ datasets as well as for the pooled data. For the discrimination task, we fitted a cumulative Gaussian function to the binary responses using the Psignifit package^18^, and estimated lapse and guess rates, the point of subjective equality (PSE), and the standard deviation of the function. The PSE corresponds to the 50% threshold of the psychometric function, and the standard deviation of the cumulative Gaussian function corresponds to the reciprocal of the psychometric function slope.

For the reproduction task, we first measured the reproduced orientation relative to the reference, $\Delta \omega$, as

1 $$\begin{aligned} \Delta \omega = {\bar{\omega }} - \omega _{ref}, \end{aligned}$$

where ${\bar{\omega }}$ was the reproduced stimulus orientation and $\omega _{ref}$ was the reference orientation, and we plotted the histogram of the estimates $\Delta \omega$ (Fig. 3). To compare the distributions of estimates between conditions quantitatively, we fitted a symmetric Gamma mixture model to the data. The model can capture and describe the potential bimodality and skewness of the distribution. The model is a mixture of two identical Gamma density functions, denoted as $\Gamma (\alpha , \theta )$, each characterized by a shape parameter $\alpha$ and a scale parameter $\theta$. The variance of each density function is thus $\alpha \theta ^2$. The model parameters were optimized by minimizing the non-linear least squares, and the optimized parameters with 95% confidence intervals were compared within participants.

To compute the repulsive bias, we selected the trials with the estimates indicating correct orientation judgment. Note that, given there was no direct measurement of judgment correctness in the single-task condition, we classified the correctness of judgment based on the subject’s estimates in the reproduction task rather than the explicit judgment responses in the discrimination task. A reproduction response was deemed correct when the estimate fell on the same CW/CCW side of the reference as the true stimulus orientation. Approximately $83.14\%$ of the total trials were selected for the analysis ($49.73\%$ were from the single-task condition, and the rest were from the dual-task condition). The repulsive bias was determined as the bias of the estimate, with a positive sign indicating repulsive bias away from the reference.

### Modeling

We used a two-component encoding-decoding model based on previous studies^10,11^, which included two main components: a measurement distribution and a weighting function. The measurement distribution represented the noisy encoding of stimulus orientation. It was modeled as a Gaussian $N (\mu , \sigma )$, with mean $\mu$ and variance $\sigma$, where $\sigma$ represented the variability of sensory representations and thus could vary between stimulus noise levels. At the decoding stage, the sensory representations were re-weighted by multiplying the measurement distribution with a weighting function. We modeled this weighting function as a symmetric gamma mixture of two identical Gamma density functions that integrate to 1:

$$\begin{aligned} f(\omega ; \alpha , \theta , \delta ) = \frac{1}{2} \cdot g(\omega ; \alpha , \theta , \delta ) + \frac{1}{2} \cdot g(\omega ; \alpha , \theta , -\delta ), \end{aligned}$$

where $\omega$ represents orientation in measurement distribution and *g* is a Gamma density function

$$\begin{aligned} g(\omega ; \alpha , \theta , \delta ) = \frac{1}{{\theta ^\alpha \cdot \Gamma (\alpha )}} \cdot (\omega - \delta )^{\alpha - 1} \cdot e^{\left(-\frac{{\omega - \delta }}{{\theta }}\right)}, \end{aligned}$$

with parameters of shape $\alpha$, scale $\theta$, and shift $\delta$, and with a Gamma function $\Gamma (\alpha )$. To capture changes due to explicit judgment at the decoding stage, we allowed for different weighting functions between two task conditions (single-task and dual-task). The model also included a constant motor bias term $\epsilon$ independent of conditions. Altogether, the model had two parameters $\sigma _l$ and $\sigma _h$ for the noise levels, six parameters defining the weighting functions ($\alpha _s$, $\theta _s$ and $\delta _s$ for the single-task condition; $\alpha _d$, $\theta _d$ and $\delta _d$ for the dual-task condition), and a motor bias term $\epsilon$.

The optimal parameters were estimated by maximizing the likelihood of measured data given the model using the Nelder-Mead algorithm^19^. All trials were included for the optimization. The optimization was performed with bootstrapping 100 times for each subject’s data and for the pooled data. Our model was designed to account for both the mean and full distribution of the participants’ estimates. Therefore, for each stimulus orientation, 500 samples were randomly drawn from the combination of the measurement distribution and the weighting function. This resulted in a set of predicted estimates, rather than mean or mode, that were analyzed in the same way as the experimental data.

## Results

### Behavioral results

To examine whether a reference orientation could bias the subject’s estimate of a preceding stimulus orientation, we analyzed and compared the estimates under different noise and task conditions.

**Figure 2:**
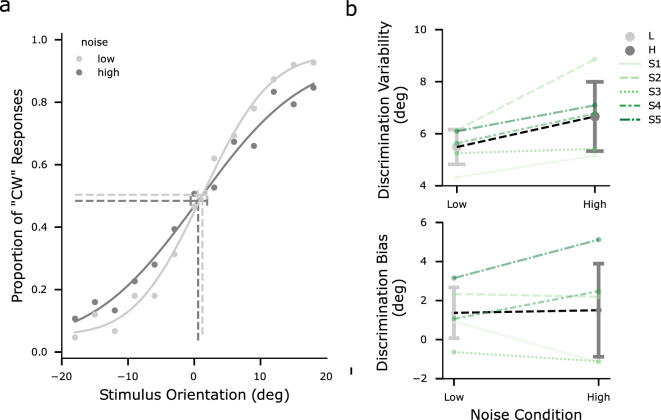
Discrimination data in the dual-task condition. (**a**) Mean proportion of clockwise (CW) responses and associated cumulative Gaussian psychometric functions, separated for low (light gray) and high (dark gray) noise levels. The x-axis represents the stimulus orientation relative to the reference orientation. Positive values indicate orientations clockwise to the reference line. Data points were pooled from the dual-task condition of all participants’ data. Vertical dashed lines denote PSEs for the two conditions. The error bar denotes one standard error of the associated PSE. (**b**) Estimated parameters of the psychometric function. Top: Discrimination variability; Bottom: Discrimination bias (PSE). Data points denote averages across all participants, error bars denote standard deviations. Dashed lines connect individual’s estimates of each noise condition.).

We first evaluated the discriminability of reference and stimulus by fitting psychometric functions to the explicit judgment responses in the dual-task condition. Discrimination data under this condition showed that all participants were able to perform the task (Fig. 2a, see Fig. S1 for individual data). For the pooled data, the discrimination threshold increased from ${5.16}^\circ \pm {0.30}^\circ$to ${6.39}^\circ \pm {0.44}^\circ$ with increasing stimulus noise levels. Likewise, stimulus noise elevated the discrimination thresholds for all individuals (sign test $p =.031$, Fig. 2b). There was no difference in the PSEs between the two noise conditions (sign test $p =.999$).

**Figure 3:**
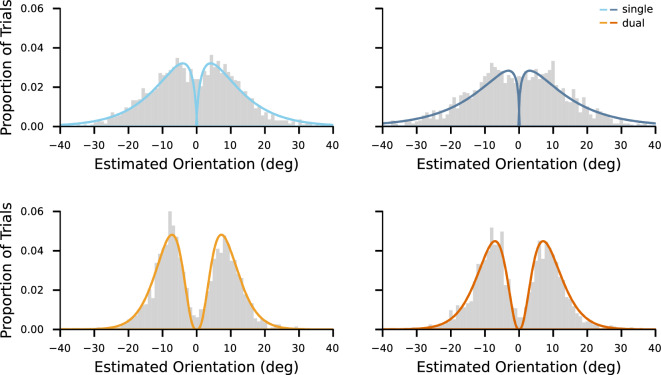
Distributions of all participants’ pooled estimates under different conditions. The x-axis represents the estimated orientation relative to the reference orientation. Curves denote the symmetric mixed Gamma density functions fitted to the distributions of estimates. The four colors of the lines represent the four conditions, where hues correspond to task conditions and shades correspond to noise levels (darker shades correspond to higher stimulus noise).

We then evaluated the bimodality of the distribution of estimates, a characteristic that previous research has identified as a crucial aspect of the reference repulsion effects^10^. Fig. 3 shows the distributions of estimates under different noise and task conditions. We applied Hartigan’s dip test of unimodality^20^ to examine the multimodality of the distributions. For the pooled data, the distribution of estimates did not show multimodality under the single-task condition (dip test $p =.120$ for low noise stimuli, $p =.682$ for high noise stimuli), while the estimates of the dual-task condition followed bimodal distributions (dip test $p <.001$ for both low and high noise stimuli).

**Figure 4:**
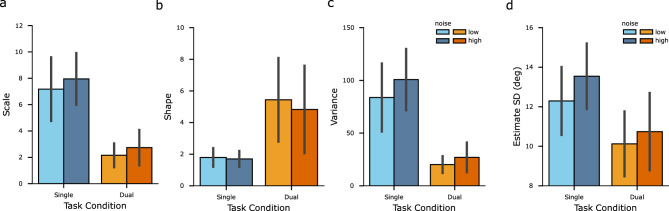
Characteristics of the distributions of estimates. (**a**–**c**) Parameters of the fitted symmetric mixed Gamma density functions. (**a**): scale parameter; (**b**): shape parameter; (**c**): derived variance. Error bars denote $\pm 1$ standard deviation across participants. (**d**) Standard deviations of participants’ estimates. Error bars denote $\pm 1$ standard deviation across participants. The four colors represent the four conditions, where hues correspond to task conditions and shades correspond to noise levels.

To further compare the distributions under the two task conditions, we fitted a symmetric mixed Gamma distribution to the distribution of estimates and subsequently compared scale ($\theta$) and shape ($\alpha$) parameters, which characterize the spread and skewness of the fit, respectively. For four out of five participants, both parameters were significantly different, with lower values of the scale parameter and higher values of the shape parameter under the dual-task condition compared to the values under the single-task condition (Fig. 4a,b, see Fig. S2 for individual’s parameters with 95% confidence intervals). This resulted in the variance of the fitted Gamma distribution, derived as $\alpha \theta ^2$, being lower under the dual-task condition than under the single-task condition (Fig. 4c). The comparison results did not depend on the specific fitting method. Similar results were obtained by fitting the distribution with a mixture of two identical Gaussian density functions or a mixture of two identical Gamma density functions with shifts.

**Figure 5:**
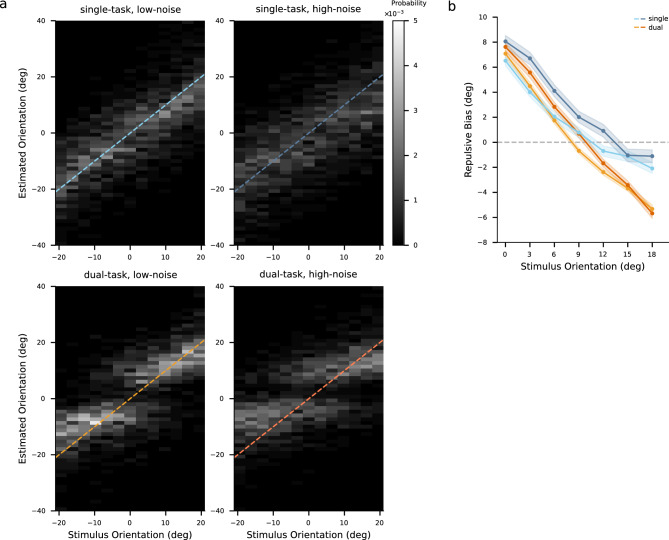
Estimates distribution and repulsive bias. (**a**) Distributions of all participants’ pooled estimates for each particular stimulus orientation. Probability is presented by gray level. Values on the x-axis and y-axis are orientations relative to the reference orientation. The dashed lines indicate where estimated orientations are equal to stimulus orientations. (**b**) Repulsive bias of all participants’ pooled data. Data are from trials where the subject’s estimates indicated that the subject correctly judged the side (CW/CCW) of the reference orientation on which stimulus orientation fell. The x-axis represents the absolute difference between the stimulus orientation and the reference orientation. Shades denote one standard error of the mean. The four colors represent the four conditions, where hues correspond to task conditions and shades correspond to noise levels.

Correspondingly, we compared the variability of all estimates under different conditions. As Fig. 4d shows (see Fig. S3 for individual data), the estimates showed larger standard deviations in the single-task condition than in the dual-task condition for most individuals (four out of five participants), while participants did not show consistent differences in standard deviations between noise conditions.

**Figure 6:**
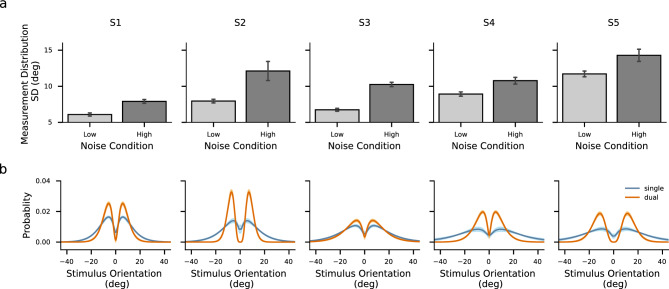
Recovered models for individual participants. (**a**) Standard deviations of estimated measurement distributions. Error bars denote $\pm 1$ standard deviation of 100 bootstrapped estimates. (**b**) Estimated weighting functions. Shaded areas around the curves denote $\pm 1$ standard deviation of 100 bootstrapped estimates.

For all participants, the estimates were biased away from the reference when the stimulus orientation was close to the reference orientation (Fig. 5a). The repulsion effect was more pronounced in the dual-task than in the single-task condition. In line with previous findings^10–12,15^, the repulsion effects weakened with increasing difference between stimulus and reference orientation (Fig. 5b, also see Fig. 8a for individual data). Stimulus noise elevated the repulsive bias of most stimulus orientations (82.8% of individual’s repulsive biases under the single-task condition and 71.4% of individual’s repulsive biases under the dual-task condition). Interestingly, for stimulus orientations with larger differences (above ${6}^\circ$) from the reference orientation, the repulsive biases were smaller under the dual-task condition compared to those under the single-task condition. Note that these repulsive biases were negative, indicating that for these stimuli the reference induced larger attraction effects under the dual-task condition compared to the single-task condition.

The overall results demonstrate that in the dual-task paradigm, participants’ estimates were biased away from a post-stimulus reference, presenting characteristic features of reference repulsion. Moreover, the distribution of these estimates was quantitatively distinct compared to those observed in tasks that did not require an explicit discrimination response.

### Modeling results

Reference repulsion has been hypothesized as a consequence of a decoding strategy in which sensory neurons are weighted according to their contributions to the discrimination between reference and stimuli. This strategy is mathematically described by an encoding-decoding model^10^. However, it remains unclear whether this model could account for the repulsion induced not only by simultaneously presented reference and the stimulus but also by a post-stimulus reference. Furthermore, the distinction between implicit and explicit discrimination and its impact on reference repulsion is still under investigation. To address these questions, we adopted an encoding-decoding model.

**Figure 7:**
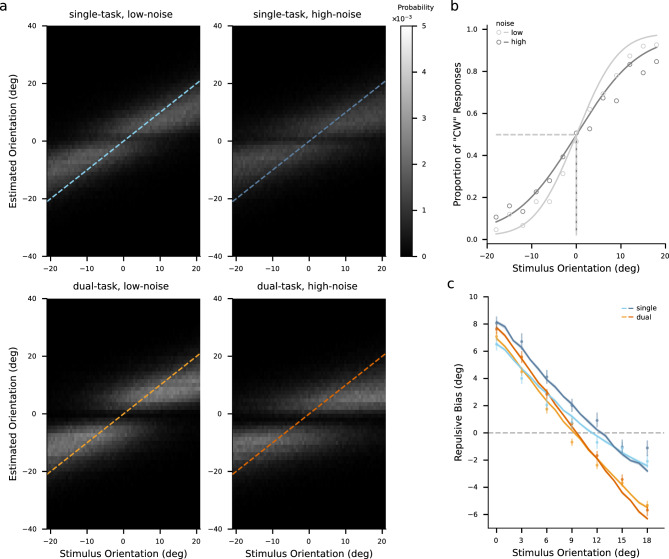
Pooled model prediction for all participants. (**a**) Distributions of estimates predicted by the estimated model for each particular stimulus orientation. The density was presented with a lightness scale. All the values on the x-axis and y-axis are orientations relative to the reference orientation. The dashed lines indicate where estimated orientations are equal to stimulus orientations. (**b**) Psychometric functions of predicted explicit judgment as “the stimulus orientation is more clockwise than the reference orientation”. The x-axis represents the stimulus orientation relative to the reference orientation. Data are from all participants’ pooled judgment responses under the dual-task condition (open circle; same as the data in Fig. 2). Solid lines denote the cumulative Gaussian function fitted to the model prediction. Dashed lines denote the $50\%$ threshold of discrimination. The error bar denotes one standard error of the estimated $50\%$ threshold. (**c**) Repulsive bias predicted by the model (solid lines). Data are repulsive bias of all participants’ pooled data (dots; same as the data in Fig. 5b). The x-axis represents the absolute difference between the stimulus orientation and the reference orientation. Shades denote one standard error of the mean of 100 bootstrapped model predictions.

**Figure 8:**
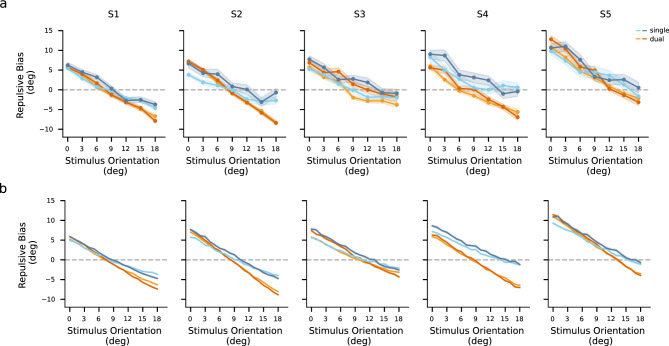
Measured and predicted repulsive bias of individuals. (**a**) Measured repulsive bias of individual participants. Data are from trials where the subject’s estimates indicated that the subject correctly judged stimulus orientation relative to the reference orientation. (**b**) Model prediction of individual’s repulsive bias. The x-axis represents the absolute difference between the stimulus orientation and the reference orientation. Shades denote one standard error of the mean. The four colors represent the four conditions, where hues correspond to task conditions and shades correspond to noise levels.

We assumed that the post-stimulus reference influences discrimination choices by re-weighting representations in working memory. In probabilistic terms, inferring an estimate involves combining a measurement distribution derived from sensory encoding with a re-weighting profile during the late stage of decision-making. Based on this assumption, we developed a two-component encoding-decoding model, consisting of a measurement distribution centered on the true stimulus orientation and a weighting function featuring a profile that was symmetrical around the reference boundary and had a mixed Gamma density function.

We derived the parameters of the model by maximizing the likelihood of the subject’s estimates. It is important to note that the variability of measurements was influenced by both external and internal noise during stimulus encoding. Consequently, we compared the measurement distributions across noise conditions. Furthermore, since the two task conditions differed in terms of whether they involved an explicit choice at a late stage, we compared the weighting functions between the conditions to investigate any potential difference. As Fig. 6a shows, the spreads of the measurement distributions increased with increasing the noise level of the stimulus (a sign test of $p <.001$). The estimated weighting functions showed different profiles between the task condition (Fig. 6b). While the averaged weighting functions among participants peaked at similar positions (around $\pm {7}^\circ$) in both task conditions, the weighting functions displayed a lower degree of concentration in the single-task condition compared to the dual-task condition for all participants, represented by larger means and broader ranges (both with a sign test of $p <.001$). The models accurately predicted the entire distribution of the estimates (Fig. 7a), the explicit judgment responses (Fig. 7b), as well as the repulsive biases (Fig. 7c, and see Fig. 8 for individual’s results).

In light of these results, we conclude that an encoding-decoding model can account for orientation estimates in the presence of a post-stimulus reference orientation, suggesting that participants use the reference-relevant information to derive an estimate by re-weighting the preceding sensory information. This re-weighting strategy appeared relevant for an explicit discrimination judgment, as evidenced by re-weighting profiles that showed a higher degree of concentration when an explicit choice was required.

## Discussion

The perception of visual stimuli is susceptible to the context in which they are presented, and specifically, judgments of a stimulus feature tend to be biased away from a reference that shares a similar feature. The present study investigated whether such repulsion effects can be induced by a reference presented after the stimulus. We found that participants’ estimates of the mean orientation of ensemble stimuli were biased away from a post-stimulus reference. These repulsive biases showed the typical characteristics of the reference repulsion effect, which indicates that the repulsive bias occurred during a late stage of visual decision-making. Moreover, the explicit discrimination judgment made by participants between the stimulus and reference impacted on the magnitude and direction of these biases. This impact can be explained by an observer model that accounted for the differential re-weighting of sensory representations between implicit and explicit processes.

### A novel paradigm with a post-stimulus reference

Our experimental paradigm complements previous studies that used a reference simultaneously presented with the stimulus [9-11]. Instead, we employed a post-stimulus reference, which we demonstrated can induce repulsion effects on the judgment of the preceding stimulus. It is important to note that while the reference influenced the participant’s estimates of the preceding stimulus, it is unlikely to reflect backward masking effects induced by the reference^21^. In contrast to the brief stimulus presentations (typically less than 50 ms) that have been used to induce backward masking effects^22^, the stimulus in our study had a presentation duration of 500 ms, which was sufficiently long for conscious visual processing of the stimulus.

Similarly, it is unlikely that the stimulus biased the perception of the subsequent reference. If the reference was systematically biased by the stimulus, we would expect consistent biases across individuals and noise conditions in the discrimination data. However, this was not observed (Fig. 2b). Moreover, considering that reference repulsion effects typically involve working memory^15^, it is unlikely that the reference presented throughout the tasks would be memorized and then biased by the stimulus. Nevertheless, our results are based on the comparison of reference-induced repulsion effects across the two task conditions. Even if any of the effects considered above were present, they would have been consistent across both conditions and thus would not substantially influence our conclusions.

### Reference repulsion as a late decision-related bias

Our findings show that the presence of a reference during the target stimulus presentation is not necessary for reference repulsion effects to occur. This provides compelling evidence that reference repulsion bias can be a late decision-related bias^11,12,14,15^, as opposed to an exclusive early-stage bias resulting from encoding or even decoding at the time of stimulus presentation^10,13^, particularly when considering the impact of the post-stimulus reference on the reproduction of a preceding stimulus.

Thus, our findings align with existing theories on information integration during late-stage perceptual inference processes^11,12,15^. Specifically, our dual-task results agree with the self-consistency theory, which posits that categorical judgments generate top-down expectations that serve as a categorical prior^12,15^. Our results extend this theory by suggesting that these categorical priors can not only reflect expectations before the exposure to sensory evidence, but can also be formed in a subsequent event, updating sensory representations in working memory.

Our results suggest that the reference repulsion effect results from the combination of contextual information and sensory representations in the perceptual inference process. However, it remains unclear whether this combined probabilistic information directly replaced the representation in working memory. According to Luu & Stocker^15^, categorical judgments introduce biases in a downstream process from working memory. In their study, participants flexibly recombined probabilistic information in working memory recall, based on feedback about their categorical judgments, maintaining self-consistency. Further research could explore whether judgments directly modify working memory representations, with a particular focus on the necessity of explicit judgment in these interactions.

### Biases in the dual-task condition

Our results showed that reference repulsion displayed the typical characteristics previously reported, such as consistency with preceding discrimination choices, increased biases for stimulus orientation closer to the reference, and a systematic influence from stimulus noise^10^. In the dual-task condition, our findings clearly aligned with these characteristics, while we also found that, for some subjects, the estimates for the stimulus orientation dissimilar to the reference displayed a greater attraction towards the reference than previously reported^12,15^.

To examine whether these attraction biases resulted from the post-stimulus reference, we conducted a control experiment with two participants under the dual-task condition. Unlike the main experiment, where the reference was presented after the stimulus, in this control experiment, the reference and the ensemble stimulus were presented simultaneously. For the participant who exhibited fairly large attraction biases in the main experiment (S1), the simultaneous presentation significantly reduced the attraction biases (ANOVA tests $p <.001$ for all stimuli with a distance larger than ${9}^\circ$). However, there was no significant difference between the main and control experiments for the other participant (S3), likely due to this participant displaying relatively small attraction biases (Fig. S4). Therefore, it is plausible that the post-stimulus reference introduced greater attraction compared to a reference presented simultaneously with the stimulus, as participants may rely more on decision categories when sensory representations decayed in working memory^23^.

An alternative explanation for the attraction bias is that participants might form and utilize prior expectations regarding the range of the stimuli^24,25^. This notion is supported by the observations of attraction biases when participants were given explicit cues about the stimulus range (see Experiment 2 in^12^). In our study, we chose the reference orientations from eight fixed values, leading to a less stochastic stimulus sampling compared to previous studies^10,12^. As participants were exposed to these stimulus orientations across trials, they may have become familiar with the statistical regularities of the reference orientations and implicitly learned that the stimulus orientations consistently fell within a certain CW/CCW range ($\pm {18}^\circ$) around the reference. Consequently, participants may have formed prior beliefs corresponding to the stimulus range, resulting in estimates being attracted toward the center of these expectations (i.e. around ${9}^\circ$ CW/CCW to the reference).

### Biases in the implicit process

We observed reference repulsion effects even in the absence of an explicit task directly related to the reference. This distinguishes our study from previous studies that employed an explicit discrimination task in the paradigm^10,12^. Instead, we employed a paradigm where the reference was only presented after the target stimulus, avoiding any contextual modulation of the reference during stimulus encoding, which excludes the classic tilt effect^3^ that occurs when the stimulus and reference are presented simultaneously, or the tilt after-effect if the reference precedes the target stimulus. Therefore, our results provide further confirmation of earlier evidence of repulsion biases that arise during passive viewing of the reference and involve the implicit processing of reference-related information^11,14,15^.

Similarly, implicit repulsion effects have also been observed in studies focusing on working memory^26–29^. Although these studies did not explicitly employ a stimulus as a reference, participants implicitly compared the memorized stimuli, resulting in repulsive interactions. Various theories have attempted to explain these repulsion effects. For example, Ding et al.^28^ suggested that the heightened difference between the stimuli yielded a temporal repulsion effect when participants performed a successive reproduction task involving two orientations. These findings indicate that the implicit use of ordinal information constrains the decoding of working memory representations. Alternatively, an adaptive perspective of inter-item bias proposes that repulsion occurs between similar stimuli^27,29^, while attraction occurs between dissimilar stimuli, thereby balancing accurate and distinct representations.

Despite the diversity of interpretations and methodologies, previous studies, in line with our findings, consistently demonstrate implicit repulsive exaggeration between similar stimulus representations in working memory. In our study, even though the reference was not a memorized item, these exaggerations may have helped participants to implicitly avoid overlapping representations.

### Comparison between the single-task and dual-task conditions

We found distinct reference repulsion effects between the two task conditions and proposed a re-weighting account that differentiated between implicit and explicit processes as a plausible explanation for the observed behavior. However, it is important to consider alternative explanations for these differences.

One possibility is that, during the reproduction phase of the dual-task condition, participants intentionally adjusted the probe further away from the reference due to the forced discrimination choice. This adjustment may have exaggerated the orientation difference between the stimulus and the reference. Such a strategy could be adopted to maintain the participant’s self-consistency^12^ when the difference between the stimulus and the reference was too small or near zero. Consistent with this expectation, we found that the magnitude of the repulsion bias when the stimulus orientation aligned with the reference orientation (${7.35}^\circ \pm {2.47}^\circ$, averaged across subjects and noise levels in the dual-task condition) matched the just noticeable difference (${7.37}^\circ \pm {2.11}^\circ$, averaged across subjects and noise levels) in the discrimination task. However, this explanation fails to account for the attraction bias towards the reference observed for stimuli with orientations that were significantly different from the reference orientation.

Another difference between the two task conditions is the memory delay before the reproduction phase. If the stimulus representations were held in working memory until the reproduction phase and read out specifically for the reproduction task, one would expect a larger variance in participant’s estimates due to larger internal memory-related noise^30–32^. However, our results do not support this hypothesis. In fact, the total time to complete was not significantly different between the dual-task and the single-task (repeated measure ANOVA, $p=.025$, see Fig. S5). Moreover, participants spent more time with the high noise stimulus when the task involved explicit judgment (interaction between noise and task conditions, repeated measure ANOVA $p=0.002$). Additionally, the reaction times were shorter under the dual-task condition than the single-task condition for most participants, although a repeated measure ANOVA test showed a significant difference only for the interaction between the task and noise conditions ($p =.025$). These comparisons of the reaction times suggest that the explicit discrimination response sped up the following reproduction task, indicating that the readout of the stimulus representation occurred at the first relevant task. Thus, the memory decay account cannot fully explain our results. However, it remains unclear whether the readout would be equivalent in terms of representation quantities and neural mechanisms to the readout for the following accurate reproduction.

Furthermore, it is also unlikely that the differential effects observed are attributable to learning over experiment blocks and task conditions. Perceptual learning has been shown to occur over trials and blocks, reducing perceptual variability^33–35^. Therefore, one might expect a decrease in the variance of the estimates over blocks, especially considering the possibility that participants learned to use the reference. Data from two participants who repeated the experiment under the single-task condition after completing the main experiment (Fig. S6) showed sharp transitions between the dual-task and single-task blocks, suggesting that the variability of the participants’ estimates was immediately reduced by the explicit discrimination, rather than gradually decreased over time through learning (Fig. S6a). These results also imply that participants were unlikely to learn to use the discrimination judgment before being exposed to the instruction of the explicit discrimination task. Interestingly, the effects of explicit discrimination were reversible and anchored to the relevant task for both participants, evidenced by no significant difference in the standard deviations of estimates between the first and repeated single-task conditions (Levene’s test $p >.05$ for both participants, see Fig. S6b).

Therefore, our results strongly support genuine distinctions between explicit and implicit processes, which may reflect the employment of different strategies for utilizing sensory information in visual perception, depending on the availability of categorical context. The re-weighting model we adopted is not limited to early visual processing or specific behavioral paradigms^10^, suggesting a fundamental disparity in information utilization between explicit and implicit processes, which may apply to other findings on sensory information processing. Studies across various topics have reported non-normative patterns of information integration in explicit processes. For example, in visual search, Hansmann et al.^16^ found that the explicit encoding of ensemble representations is based on summary statistics, whereas the implicit assessment encodes ensembles with rich details. Similarly, Chen et al.^36^ found differences in the use of category information between implicit and explicit processes in category-based induction. When predicting the direction of moving geometric figures that were categorized by learning, participants were more likely to integrate information across categories in the implicit process, whereas the explicit process was dominated by single-category information. Consistent with these theories, our model suggests that the explicit process prioritizes the coarse information about statistical and categorical representations, while the implicit process tends to utilize rich sensory representations.

Finally, the role of explicit choices in contextual information processing remains an open question. Explicit choices may lead to feature-oriented attention and allocate more cognitive resources to the reference^37^. Top-down attentional guidance directs limited cognitive resources towards task-relevant signals for optimal performance^38,39^. Therefore, it is possible that in the present study, participants paid more attention to the reference in the dual-task condition compared to the single-task condition, as they were aware of its relevance to the task. This increased attention to the reference could potentially result in distinct biases between the two conditions. Moreover, explicit choices may alter decision-making, suggested by a recent study showing that explicit choices induce the down-weighting of late evidence in the accumulation of decision-relevant information, which is reflected by pupil-linked arousal^7^. Another possibility is that explicit choices increase the gain of neurons encoding the reference, resulting in contextual modulation of the representation of the stimulus in working memory. Population coding models have extensively explained contextual biases, which posit modulations on the responses of neuronal populations in the visual cortex^6,40–44^. For example, neurons in population codes may adjust their gains to different degrees as a consequence of the context, leading to biases^42,44^. It is conceivable that explicit choices increase the gain around the reference and thus enhance the repulsion effect. However, the traditional population coding model has limitations in explaining the reference-induced attraction effect. Our results suggest a strategy for extending the model to account for the effects of explicit choices by incorporating the reweighting components we identified.

## Conclusion

Reference repulsion is a well-known phenomenon that demonstrates how contextual information influences visual perception. Such repulsion may occur at an early, sensory-related stage, or a late, decision-related stage of visual processing. To investigate the influence of the reference during late visual processing, we conducted an experiment using an ensemble stimulus of orientation, followed by the presentation of a reference orientation. We found strong repulsion effects induced by the post-stimulus reference, as evidenced by the significant bias in participants’ reported stimuli. Moreover, the explicit discrimination made between the reference and the stimulus had a notable impact on the magnitude of repulsion effects. This impact can be effectively explained by an encoding-decoding model that differentiates the re-weighting of sensory representations between implicit and explicit processes. In summary, our findings provide evidence that reference repulsion can occur during late visual processing, indicating distinct sensory decoding between implicit and explicit tasks.

### Supplementary Information

Supplementary Figures.

## Data Availability

The data and code can be found at the G-Node GIN platform: 10.12751/g-node.46h2gl.
