# Supplementary material for: Reference induces biases in late visual processing
Source: Sci Rep. 2023 Oct 30;13:18624. doi: 10.1038/s41598-023-44827-8 (PMC10616182; doi:10.1038/s41598-023-44827-8)
Supplement: Supplementary file 1 — Supplementary Figures. [file 41598_2023_44827_MOESM1_ESM.pdf]

Reference induces biases in late visual processing

Yannan Su[1,2*], Thomas Wachtler[1,3], and Zhuanghua Shi[4]

[1]Faculty of Biology, Ludwig-Maximilians-Universität München, Munich, Germany
[2]Graduate School of Systemic Neurosciences, Ludwig-Maximilians-Universität München, Munich, Germany
[3]Bernstein Center for Computational Neuroscience, Munich, Germany
[4]General and Experimental Psychology, Ludwig-Maximilians-Universität München, Munich, Germany
Email: su@biologie.uni-muenchen.de

Supplementary Information

Supplementary Methods

Two of five participants completed supplementary experiments after completing the main experiment.

To examine any potential learning effect, we asked two participants to repeat the experiment under the single-task condition after the main experiment. The stimuli and procedures were the same as those in the single-task condition of the main experiment.

To measure the repulsion effect from a reference that is presented throughout the experiment trial, we asked two participants to repeat the experiment under the dual-task condition. The stimuli and procedures remained compared to those in the dual-task condition of the main experiment, while the reference was presented simultaneously with the ensemble and lasted until the final response.

Supplementary Figures

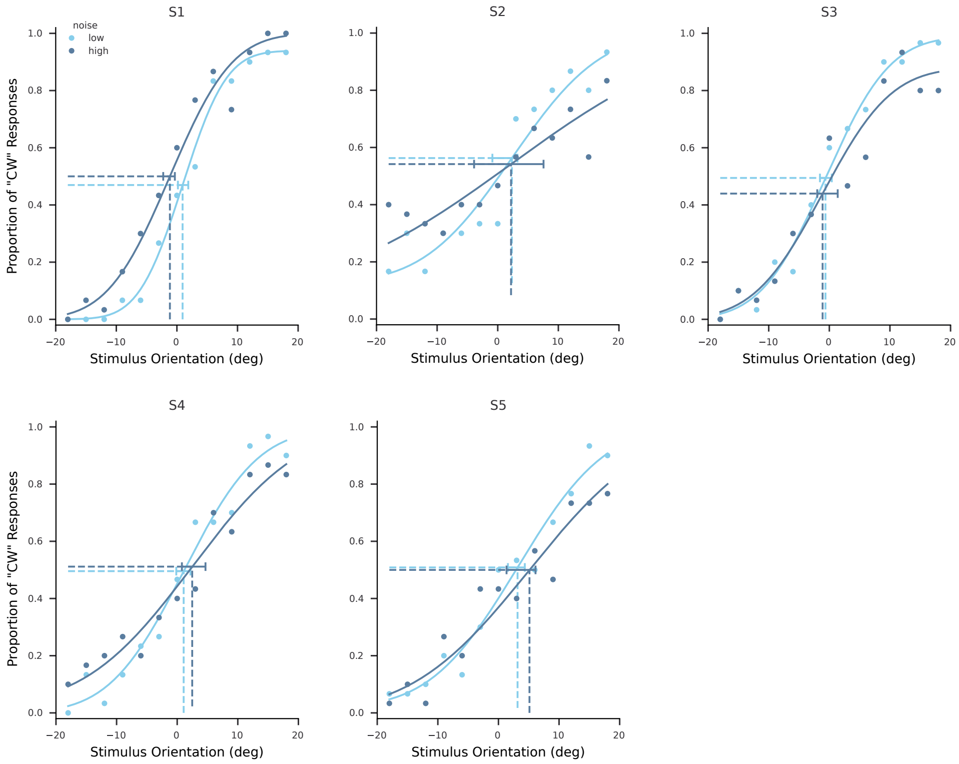

Figure S1: Individual's discrimination data in the dual-task condition: mean proportion of clockwise (CW) responses and associated cumulative Gaussian psychometric functions, separated for the low and high noise levels. The x-axis represents the stimulus orientation relative to the reference orientation. Positive values mean the orientation clockwise to the reference line. Data points were pooled from the dual-task condition of all participants' data. Vertical dashed lines denote PSEs for two conditions respectively, and the error bar denotes one standard error of the associated PSE.

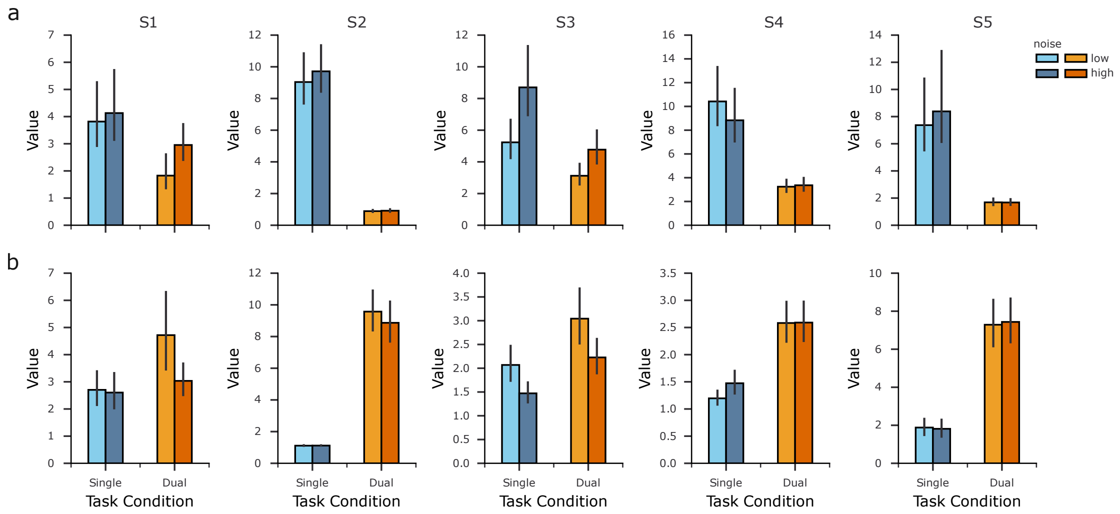

Figure S2: Estimated parameters of fitted symmetric mixed Gamma density functions for individuals. (a): scale parameter; (b): shape parameter. Error bars denote 95% confidence intervals of the estimates. The four colors represent the four conditions, where hues correspond to task conditions and shades correspond to noise levels.

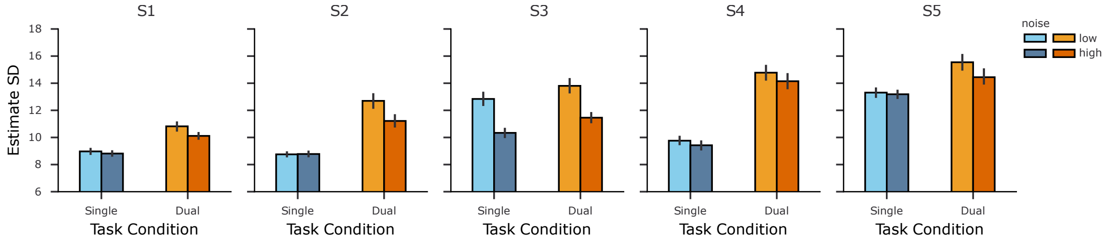

Figure S3: Standard deviations of individual's estimates. Error bars denote 95% confidence intervals of 100 bootstrapping resamples. The four colors represent the four conditions, where hues correspond to task conditions and shades correspond to noise levels.

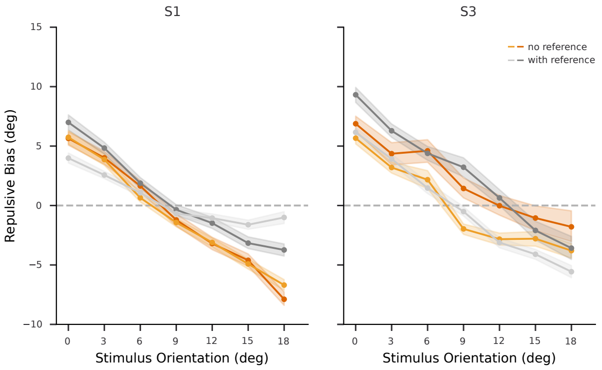

Figure S4: Repulsive bias of two participants' estimates. Data are from trials where the subject's estimates indicated that the subject correctly judged stimulus orientation relative to the reference orientation. The orange colors represent the data under the dual-task condition in the main experiment, where the reference was absent in the stimulus presentation. The gray colors represent the data of a control experiment where the reference was present simultaneously with the stimulus in the dual-task condition. The shades of colors correspond to the noise level. The x-axis represents the absolute difference between the stimulus and reference orientations. Shades denote one standard error of the mean.

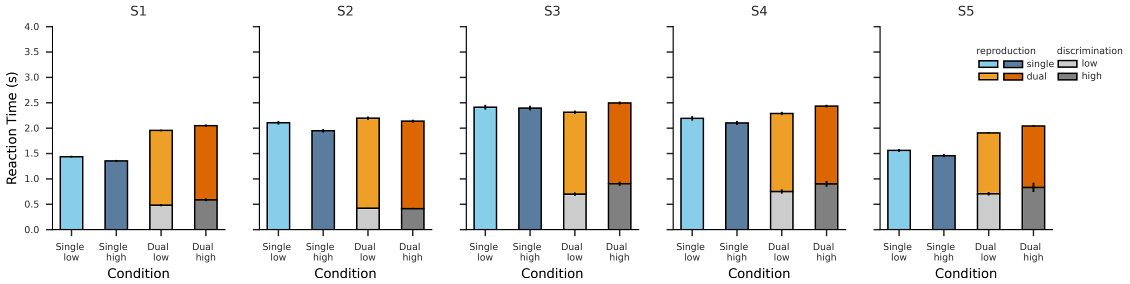

Figure S5: Individual's reaction times for all tasks. The four blue and orange colors represent the four conditions of reproduction reaction time, where hues correspond to task conditions and shades correspond to noise levels. The gray colors represent the noise conditions of discrimination reaction time in the dual-task condition.

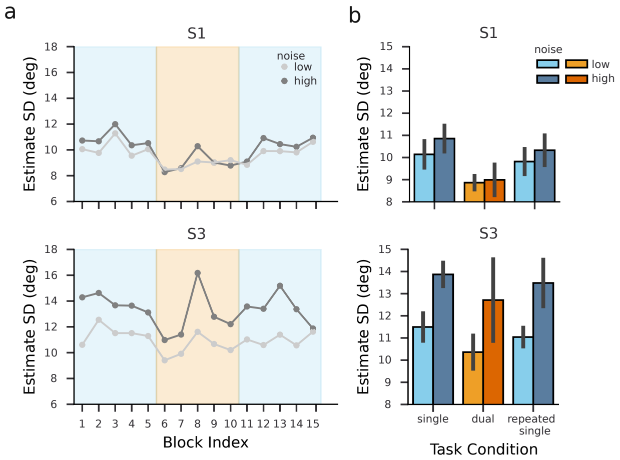

Figure S6: Variations of two participants' estimate standard deviations. (a) The standard deviations of estimates varied over blocks. The shade represents the task condition of the corresponding block (blue: single-task, orange: dual-task). (b) The standard deviations of estimates varied over task conditions. Error bars denote ± 1 standard deviation.
